# NPM-ALK: The Prototypic Member of a Family of Oncogenic Fusion Tyrosine Kinases

**DOI:** 10.1155/2012/123253

**Published:** 2012-07-18

**Authors:** Joel D. Pearson, Jason K. H. Lee, Julinor T. C. Bacani, Raymond Lai, Robert J. Ingham

**Affiliations:** ^1^Department of Medical Microbiology and Immunology, University of Alberta, Edmonton, AB, Canada T6G 2E1; ^2^Department of Laboratory Medicine and Pathology, University of Alberta, Edmonton, AB, Canada T6G 2B7

## Abstract

Anaplastic lymphoma kinase (ALK) was first identified in 1994 with the discovery that the gene encoding for this kinase was involved in the t(2;5)(p23;q35) chromosomal translocation observed in a subset of anaplastic large cell lymphoma (ALCL). The NPM-ALK fusion protein generated by this translocation is a constitutively active tyrosine kinase, and much research has focused on characterizing the signalling pathways and cellular activities this oncoprotein regulates in ALCL. We now know about the existence of nearly 20 distinct ALK translocation partners, and the fusion proteins resulting from these translocations play a critical role in the pathogenesis of a variety of cancers including subsets of large B-cell lymphomas, nonsmall cell lung carcinomas, and inflammatory myofibroblastic tumours. Moreover, the inhibition of ALK has been shown to be an effective treatment strategy in some of these malignancies. In this paper we will highlight malignancies where ALK translocations have been identified and discuss why ALK fusion proteins are constitutively active tyrosine kinases. Finally, using ALCL as an example, we will examine three key signalling pathways activated by NPM-ALK that contribute to proliferation and survival in ALCL.

## 1. The ALK Receptor Tyrosine Kinase

Anaplastic lymphoma kinase (ALK) is a receptor tyrosine kinase of the insulin receptor superfamily, and in mice and humans, the normal expression of ALK is largely restricted to the brain and nervous system [1–4]. Mice deficient in ALK appear to have no overt developmental abnormalities [5–8]; however, behavioural abnormalities have been noted in these mice. ALK-deficient mice perform better on tests of cognitive ability and display less anxiety than their wild-type littermate controls [6, 7]. Behavioural tests also demonstrated increased alcohol consumption and altered sensitivity to alcohol in ALK-deficient mice compared to wild-type littermates [8]. Intriguingly, single-nucleotide polymorphisms (SNPs) in *ALK* have been identified in humans that correlate with decreased response to alcohol [8]. A correlation between *ALK *SNPs and schizophrenia has also been noted in a Japanese study [9].

In *Drosophila melanogaster*, the jelly belly protein (Jeb) has been characterized as an ALK ligand [10–12]. In mammals, there does not appear to be a Jeb homologue but two ligands for ALK have been described, pleiotrophin [13] and midkine [14]. However, there is not complete agreement regarding whether these are indeed ALK stimulating ligands [15, 16]. More recently, Perez-Pinera and colleagues proposed an alternative mechanism by which pleiotrophin could be stimulating ALK signalling. In their model, the binding of pleiotrophin to its known receptor, receptor tyrosine phosphatase *β*/*ζ* (RPTP *β*/*ζ*), relieves the inhibitory dephosphorylation of ALK by RPTP *β*/*ζ*, thereby turning on ALK signalling [17]. ALK has also been suggested to be a dependence receptor [18]. Dependence receptors induce apoptosis in their nonliganded state, but suppress apoptotic signalling in response to ligand binding [19].

## 2. The Identification of NPM-ALK and Other ALK Fusion Proteins

ALK-positive anaplastic large cell lymphomas (ALK+ ALCL) are a distinct subset of non-Hodgkin lymphomas with a T or null cell immunophenotype recognized by the World Health Organization Classification Scheme for hematological neoplasms [58, 59]. These lymphomas express the CD30 (Ki-1) surface antigen, but the morphologic identification of ALK+ ALCL can be challenging, as the cytologic features of the tumor cells can be highly variable from case to case. Nevertheless, the identification of the so-called “hallmark cells,” which are characterized by a horseshoe- or kidney-shaped nucleus and a prominent perinuclear Golgi body, can facilitate the diagnosis [58, 59]. Regarding the pathobiology of ALK+ ALCL, several groups in the late eighties and early nineties noted that these lymphomas possessed a recurrent chromosomal translocation, the t(2;5)(p23;q35) translocation [60–64]. In 1994, it was demonstrated that this translocation generates a fusion gene between a previously uncharacterized tyrosine kinase on chromosome 2, and the *nucleophosmin* (*NPM*) gene on chromosome 5 [20, 21]. This kinase was termed ALK owing to its association with ALCL and the expression of this kinase led to the identification of what is now considered to be a clinically distinct entity, ALK+ ALCL. In addition to NPM, several other ALK translocation partners have since been identified in ALK+ ALCL [23, 24, 27, 29–31, 33, 39, 42, 43, 45]. ALK fusion proteins have also been reported in other cancers (Table 1). These cancers include a portion of inflammatory myofibroblastic tumours (IMT) [25, 32, 34, 43, 44, 46, 55, 65], nonsmall cell lung carcinomas (NSCLC) [28, 49, 54, 57], diffuse large B-cell lymphomas (DLBCL) [22, 35–37, 47, 48, 51], colon cancers [50, 56], breast cancers [50], renal cell carcinomas [26, 52, 53], and extramedullary plasmacytomas [38]. Two papers also reported detecting tropomyosin 4- (TPM4-)ALK fusion protein expression in some cases of esophageal squamous cell carcinoma [40, 41]. Moreover, it has very recently been established that inhibitors of ALK are effective at treating patients with ALK+ ALCL [66] and other malignancies expressing ALK fusion proteins [67, 68]. Although not a focus of this paper, ALK has been reported to be highly expressed in breast cancer [69], and ALK gene amplifications and activating mutations have been identified in familial and sporadic neuroblastoma [70–75] and thyroid cancer [76].

The t(2;5)(p23;q35) translocation generates a fusion gene termed *NPM-ALK* whose transcription is under the control of *NPM* regulatory sites. NPM is a ubiquitously expressed protein that is predominately found in the nucleolus [77], but can shuttle between the cytoplasm and nucleus [78]. NPM is multifunctional and regulates several cellular activities including transcription, ribosome biogenesis, and the shuttling of proteins between the nucleus and cytoplasm [79]. The *NPM-ALK* fusion gene consists of the first four exons of *NPM* which encodes for the first 117 amino acids of the NPM protein, and the *ALK* portion of the fusion includes the exons encoding for the intracellular tail and kinase domain of ALK [20]. Importantly, the NPM part of the fusion includes the NPM dimerization/oligomerization domain [80, 81]. As we will discuss in the next section, this domain is critically important for NPM-ALK activity, and the presence of a dimerization/oligomerization domain is a common feature of other ALK fusion partners.

## 3. The Importance of Dimerization/Oligomerization Domains in ALK Fusion Proteins

An essential role for the NPM portion of NPM-ALK was first demonstrated by experiments showing that deletion of the entire NPM region of NPM-ALK generated a protein incapable of transforming NIH 3T3 cells [80]. Similarly, Bischof et al. demonstrated that NPM truncation or deletion mutants were not tyrosine phosphorylated and were unable to transform Fischer Rat 3T3 cells [81]. Since NPM has been reported to form hexamers and other oligomers [82, 83], researchers examined whether NPM could be providing an oligomerization domain in NPM-ALK. Indeed, gel filtration [80] and sucrose gradient [81] experiments demonstrated that NPM-ALK forms oligomeric complexes in an NPM-dependent manner. Moreover, NPM-ALK can dimerize with endogenous NPM, and it is believed that this accounts for why some NPM-ALK is observed in the nucleus [81].

The basic domain of Echinoderm microtubule-associated protein-like 4 (EML4) also functions as a dimerization domain in EML4-ALK [49], and this is likely mediated by a coiled-coil motif within the basic domain [84]. Most other ALK fusion partners possess known dimerization/oligomerization domains that are postulated to mediate dimerization/oligomerization of the fusion proteins (Table 2). MSN-ALK (a fusion between moesin and ALK) appears not to have an oligomerization domain and is postulated to be activated through the colocalization of MSN-ALK fusion proteins to cellular membranes [42]. Thus, dimerization, oligomerization, or colocalization of ALK fusion proteins appears to be a common and necessary requirement for these oncoproteins to signal.

## 4. Signalling Pathways Activated by NPM-ALK in ALK+ ALCL

NPM-ALK activates downstream signalling events that promote proliferation, prevent apoptosis, and enhance migration in ALK+ ALCL (reviewed in [5, 85, 86]). We will focus on the STAT3, MEK/ERK, and PI3K/Akt pathways, as much is known about the role these pathways play in ALK+ ALCL pathogenesis. In particular, we will discuss the cellular processes these pathways regulate in this lymphoma, and how they are regulated by NPM-ALK signalling.

## 5. The STAT3 Pathway

**Figure 1:**
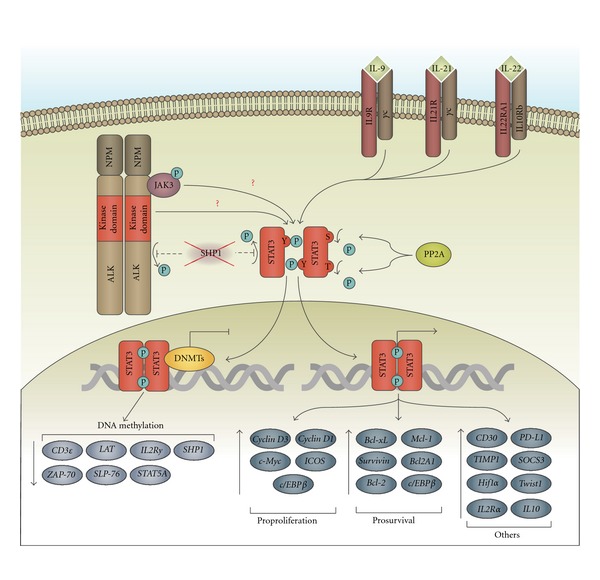
The STAT3 signalling pathway in ALK+ ALCL. STAT3 is activated by NPM-ALK signalling, but reports differ as to whether this is JAK3-dependent or independent. The phosphatase, PP2A, and signalling through the IL-9, IL-21, and IL-22 receptors also promote STAT3 activation in ALK+ ALCL. STAT3 promotes the expression of genes that suppress apoptosis and enhance proliferation in ALK+ ALCL. STAT3 can also repress a variety of genes in this malignancy through DNA methylation. Suppression of the SHP1 phosphatase by STAT3 is particularly important in ALK+ ALCL, as SHP1 inhibits NPM-ALK and STAT3 activity.

Members of the signal transducer and activator of transcription (STAT) family of transcription factors are activated by interferon, cytokine, and growth factor receptor signalling [87]. The tyrosine phosphorylation of STATs by tyrosine kinases, particularly the Janus kinases (JAKs), facilitates the dimerization of STATs. This allows the STATs to translocate to the nucleus and promote the transcription of genes involved in proliferation, cell survival, and the immune response [87, 88]. In ALK+ ALCL, the activation of STAT3 has been strongly implicated in the pathogenesis of this lymphoma (Figure 1).

STAT3 is activated in ALK+ ALCL cell lines and patient samples [89–91], as well as cells isolated from NPM-ALK transgenic mice [92, 93], as measured by its phosphorylation on tyrosine 705. The inhibition of STAT3 in ALK+ ALCL cell lines, either through the overexpression of a dominant-negative STAT3 construct [94] or decreasing STAT3 expression using antisense oligonucleotides [93], resulted in decreased proliferation and the induction of apoptosis. STAT3 was also required for NPM-ALK to transform mouse embryo fibroblasts, and for the continued survival of T-cell lymphomas induced in mice by the expression of an NPM-ALK transgene [93].

STAT3 exerts its biological effects in ALK+ ALCL through regulating the expression of multiple genes. Microarray studies performed by Piva and colleagues demonstrated that knockdown of STAT3 altered the expression of ~1500 genes in a variant of the SUP-M2 ALK+ ALCL cell line [95]. Importantly, STAT3 functions both as an activator and repressor of transcription, and approximately 60% of the STAT3-regulated genes identified by Piva et al. were repressed by STAT3 [95]. Several additional studies have identified STAT3 regulated genes in ALK+ ALCL. Those genes found to be upregulated by STAT3 include: genes that promote proliferation such as *Cyclin D1, Cyclin D3, c-Myc, ICOS, C/EBP*β** [93–97]; those that promote survival such as *Bcl-xL, Survivin, Bcl-2, Mcl-1, Bcl2A1, C/EBP*β** [90, 93, 97, 98]; others including *CD30, PD-L1, TIMP-1, Socs3, Hif1*α*, Twist1, IL10, and IL2R*α** chain [94, 95, 99–104]. STAT3 is also responsible for repressing the expression of T-cell genes that are commonly not expressed in ALK+ ALCL including *CD3*ε**, *ZAP-70*, *LAT*, and *SLP-76*, and it appears to do so in part through the upregulation of DNA methyltransferases (DNMTs) [105]. DNMTs methylate CpG motifs in promoter regions of genes, and this blocks the binding of some transcription factors and facilitates the recruitment of Methyl-C binding proteins to these promoters. methyl-C binding proteins can then recruit histone deacetylases and methyltransferases that convert promoter regions into transcriptionally inactive heterochromatin [106]. Zhang and colleagues demonstrated that STAT3 also promotes the binding of DNMTs 1–3 to the IL2R*γ* promoter in order to repress *IL2R*γ** gene expression [107]. Silencing IL2R*γ* chain expression appears to be critical in ALK+ ALCL as re-introduction of the IL2R*γ* into ALK+ ALCL cell lines resulted in decreased NPM-ALK expression and reduced viability [107]. This study also demonstrated that STAT3 enhances DNMT1 expression through the suppression of the DNMT1-targeting microRNA, miR-21. STAT3 is also responsible for the epigenetic silencing of STAT5A in ALK+ ALCL, which prevents STAT5A from repressing NPM-ALK expression and thereby interfering with NPM-ALK signalling [108]. Given the importance of STAT3 transcriptional activity in ALK+ ALCL, it is not surprising that many mechanisms contribute to the activation of STAT3 in this lymphoma.

STAT3 [89, 109] and JAK3 [90] have both been shown to coimmunoprecipitate with NPM-ALK, and several studies have shown that NPM-ALK promotes the tyrosine phosphorylation of STAT3 [89, 90, 92, 93]. However, there is not complete agreement regarding whether STAT3 tyrosine phosphorylation is JAK3 dependent [94, 110], or whether STAT3 is tyrosine phosphorylated in a JAK3-independent manner, possibly through direct tyrosine phosphorylation by NPM-ALK [111]. The serine/threonine phosphatase PP2A has also been implicated in positively regulating STAT3 activity in ALK+ ALCL, as inhibition of PP2A activity with Calyculin A was demonstrated to reduce STAT3 tyrosine phosphorylation [89]. STAT3 signalling is also likely enhanced in this lymphoma due to the fact that ALK+ ALCL cell lines do not express the STAT3 inhibitor, PIAS3 [89]. Moreover, the SHP-1 tyrosine phosphatase is often silenced by DNA methylation in ALK+ ALCL [112, 113], and this is likely due in part to the recruitment of DNMTs and histone deacetylases to the SHP-1 promoter by STAT3 [113]. Silencing SHP-1 in ALK+ ALCL is important as SHP-1 negative regulates NPM-ALK signalling through either the direct or indirect dephosphorylation of NPM-ALK, JAK2, and STAT3 [114–116], and the targeting of NPM-ALK for proteasomal degradation [115, 116]. 

Cytokine signalling also plays a role in regulating STAT3 activity in ALK+ ALCL. Signalling through the IL9 [117], IL21 [118], and IL22 [119] receptors has been shown to promote STAT3 activation in this lymphoma, and much of this may be due to autocrine signalling. Furthermore, the expression of the IL22R1 subunit of the IL22 receptor is promoted by NPM-ALK, demonstrating a link between NPM-ALK and cytokine signalling in this lymphoma [119]. Since the IL9 and 21 receptors utilize the IL2R common *γ* chain, these findings still need to be reconciled with the results of Zhang and colleagues which found that the IL2R*γ* chain is silenced in ALK+ ALCL [107].

## 6. The MEK/ERK Pathway 

Signalling mediated by the extracellular signal-regulated kinases 1 and 2 (ERK1 and 2) promotes proliferation, survival, differentiation, and migration [120]. These serine/threonine kinases are activated by many growth factor receptors through a well-defined kinase cascade. This kinase cascade is initiated by the activation of the Ras GTPase, which activates the Raf-1 serine/threonine kinase. Raf-1 then activates the dual specificity kinases, MAPK/Erk kinases 1 and 2 (MEK1 and 2), which phosphorylate and activate the ERKs [121].

**Figure 2:**
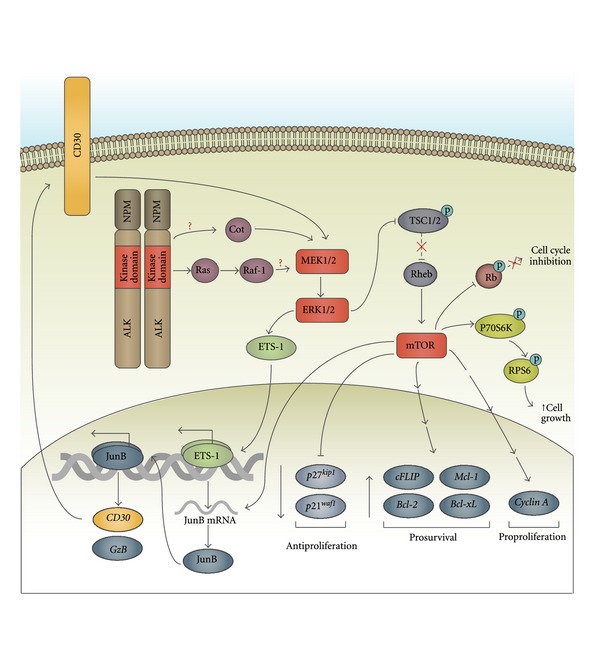
The MEK/ERK signalling pathway in ALK+ ALCL. NPM-ALK activates Ras, Raf-1, MEK1/2, and ERK1/2. The ability of NPM-ALK to activate MEK/ERK appears not to be dependent on Raf-1. Rather, another MAP3K, Cot, may be important for activation of MEK/ERK in ALK+ ALCL, but it is not known whether Cot is activated by NPM-ALK signalling. The activation of ERK1/2 promotes ALK+ ALCL proliferation and survival, largely through the JunB transcription factor and serine/threonine kinase, mTOR. ERK1/2 activates the ETS-1 transcription factor which promotes the transcription of *JunB*. JunB promotes the transcription of *CD30* and *Granzyme B* in this lymphoma, but likely has other important targets that have not yet been identified. ERK1/2 are thought to activate mTOR signalling in ALK+ ALCL by phosphorylating and inhibiting TSC1/2. mTOR phosphorylates and inhibits the cell cycle inhibitor, Rb. It also phosphorylates and activates p70S6K which phosphorylates RPS6 to promote cell growth. mTOR also influences the expression of genes that contribute to the survival and proliferation of ALK+ ALCL cells. MEK/ERK are also activated by signalling through CD30 in ALK+ ALCL, and this leads to enhanced CD30 expression.

The ERK pathway is activated in ALK+ ALCL cell lines and patient samples [122, 123] and plays a central role in promoting cell proliferation and suppressing apoptosis in this cancer (Figure 2). Treatment with the MEK1/2 inhibitor, U0126, was found to reduce proliferation [123–125] and enhance apoptosis [124, 125] in ALK+ ALCL cell lines. Reduced proliferation was also evident when the Karpas 299 ALK+ ALCL cell line was treated with ERK1 and/or 2 siRNA [124]. However, only the silencing of ERK1 in these cells was found to increase apoptosis [124]. Two important downstream mediators of MEK/ERK signalling in ALK+ ALCL are the serine/threonine kinase, mammalian target of rapamycin (mTOR), and the JunB transcription factor.

The mTOR pathway has been demonstrated to be activated in ALK+ ALCL patient samples, as measured by phosphorylation of mTOR [125, 126] and downstream targets of mTOR signalling [123, 125–127]. Marzec and colleagues found that treatment of the SU-DHL-1 ALK+ ALCL cell line with MEK inhibitors or ERK1/2 siRNA resulted in reduced phosphorylation of the ribosomal S6 protein (RPS6) [127]. RPS6 is a downstream target of mTOR signalling, and phosphorylation of RPS6 promotes cell growth [128]. The p70 S6 kinase (p70S6K), which is activated by mTOR and phosphorylates RPS6, is also inhibited in SU-DHL-1 cells treated with U0126 [129], but surprisingly not in the SR-786 ALK+ ALCL cell line [123]. MEK/ERK signalling was postulated to activate mTOR through inhibition of the tuberous sclerosis complex (TSC) [127]. TSC is a GTPase-activating protein that inhibits mTOR through inactivating the Rheb GTPase [130]. The notion that MEK/ERK signalling inhibits TSC in ALK+ ALCL is supported by the finding that treatment of SU-DHL-1 cells with MEK inhibitors resulted in decreased phosphorylation of TSC2 on inhibitory serine 1798 [127]. The activation of mTOR and the phosphorylation of mTOR substrates, eukaryotic initiation factor 4E-binding protein-1 (4E-BP1) and p70S6K, has also been demonstrated to be dependent on PI3K and Akt activity in ALK+ ALCL [126]. Phosphorylation of 4E-BP1 by mTOR results in the dissociation of 4E-BP1 from eukaryotic initiation factor 4E (EIF4E), which allows EIF4E to initiate translation [131]. However, the importance of the PI3K/Akt pathway in the activation of mTOR in ALK+ ALCL has been questioned [127].

Treatment of ALK+ ALCL cell lines with the mTOR inhibitor, rapamycin, resulted in reduced proliferation [123, 125–127, 129] and the induction of apoptosis [126, 127]. siRNA-mediated knockdown of mTOR was similarly found to reduce proliferation and enhance apoptosis in ALK+ ALCL cell lines [126]. Decreased proliferation as a result of mTOR inhibition is at least in part due to the dephosphorylation of the retinoblastoma (Rb) protein, decreased Cyclin A expression, and increased expression of the cyclin-dependent kinase inhibitors, p27^*kip*1^ and p21^*waf*1^ [126]. Increased apoptosis in response to rapamycin treatment is likely due to decreased expression of the antiapoptotic proteins Bcl-2, Bcl-xL, Mcl-1, and c-FLIP [126]. Inhibition of mTOR was also demonstrated to reduce the size of NPM-ALK-expressing murine tumours in immunocompromised mice [132].

The transcription of JunB is also promoted by MEK signalling in ALK+ ALCL cell lines [122, 123], through the ETS-1 transcription factor [133]. Interestingly, mTOR signalling also contributes to enhanced JunB translation in ALK+ ALCL cell lines through the targeting of *JunB* mRNA to ribosome-rich polysomes [123]. JunB is an AP-1 family transcription factor that is highly expressed in ALK+ ALCL cell lines and patient samples [134–136] and has been shown to promote the proliferation of the Karpas 299 ALK+ ALCL cell line [123]. JunB also influences phenotypic characteristics of this lymphoma through promoting the transcription of CD30 [122, 137] and the Granzyme B serine protease [138]. CD30 signalling also activates MEK/ERK/JunB signalling in this lymphoma to further promote CD30 expression [122].

The activation of Raf-1, MEK, and ERK in ALK+ ALCL cell lines is dependent on NPM-ALK activity [124, 139], and the ectopic expression of NPM-ALK has also been demonstrated to induce the activation of these proteins [123, 124, 140, 141]. NPM-ALK can activate Ras when ectopically expressed in the Jurkat T leukemia cell line, and the expression of a dominant negative N17 Ras decreased NPM-ALK-dependent NF-AT/AP-1 luciferase activity in Jurkat cells. Furthermore, treatment of the SU-DHL-1 ALK+ ALCL cell line with the Ras inhibitor, FTI-277, resulted in increased apoptosis and decreased proliferation [125]. Several mechanisms for how NPM-ALK activates Ras have been postulated. The Ras activator, Son of Sevenless (SOS), has been argued to be recruited to NPM-ALK via the adapter protein Grb2 by molecules such as Shc, SHP2, and insulin receptor substrate-1 (IRS-1) [80, 141–143]. Ras activation has also been proposed to occur through a PLC*γ*-dependent activation of Ras guanyl nucleotide-releasing protein (RasGRP) [80]. While Raf-1 is activated by NPM-ALK, it does not appear to be required for ERK activation in ALK+ ALCL cell lines [124]. Another MAP3K, Cot/MAP3K8, may be the primary activator of MEK in this lymphoma. Treatment of the SU-DHL-1 cell line with Cot siRNA or a Cot inhibitor decreased ERK and mTOR activation and reduced cellular proliferation [129]. Whether Cot is regulated by NPM-ALK was not investigated in this study.

## 7. The PI3K/Akt Pathway

The phosphatidylinositol 3′-kinase (PI3K)/Akt pathway regulates cell growth, differentiation, apoptosis, metabolism, and migration [144, 145]. PI3K is composed of two subunits, a regulatory p85 subunit and a catalytic p110 subunit, and this enzyme phosphorylates inositol phospholipids on the 3′ position of the inositol ring [144, 145]. These lipids, in turn, activate a number of Pleckstrin Homology (PH) domain-containing proteins; most notably the serine/threonine kinase Akt [144, 145].

**Figure 3:**
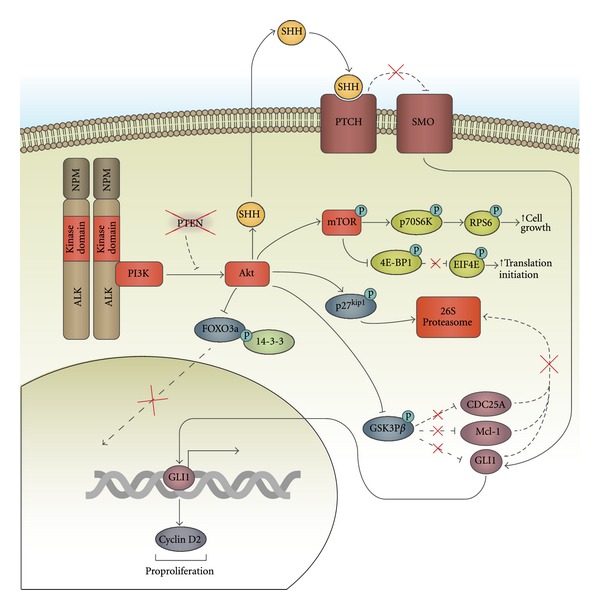
The PI3K/Akt signalling pathway in ALK+ ALCL. NPM-ALK associates with and activates PI3K, which, in turn, activates the serine/threonine kinase Akt. Expression of the PTEN lipid phosphatase, which inhibits PI3K signalling, is lost in some ALK+ ALCL tumour samples and likely contributes to Akt activation in cancers where PTEN is not expressed. Akt inhibits GSK3*β* activity in ALK+ ALCL, which protects GLI1, Mcl-1, and CDC25A from proteasomal degradation. Akt also phosphorylates the cell-cycle inhibitor, p27^*kip*1^, in ALK+ ALCL and this results in the targeting of p27^*kip*1^ for proteasomal degradation. Phosphorylation of the FOXO3a transcription factor by Akt results in the binding of FOXO3a to 14-3-3 proteins. This sequesters FOXO3a in the cytoplasm, preventing it from translocating to the nucleus and transcribing pro-apoptotic and cell cycle inhibitory genes. In addition to being an important downstream target of MEK/ERK signalling in ALK+ ALCL, mTOR activity may also be promoted by PI3K/Akt signalling. NPM-ALK/Akt signalling also promotes the expression of SHH. When SHH binds its receptor, Patched (PTCH), this relieves the inhibition of the Smoothened (SMO) coreceptor by Patched. This allows Smoothened to activate the GLI1 transcription factor, which promotes the transcription of the proproliferation protein, *Cyclin D2*.

Signalling through the PI3K pathway promotes cell survival and proliferation in ALK+ ALCL (Figure 3). Treatment of ALK+ ALCL cell lines or Ba/F3 cells ectopically expressing NPM-ALK with PI3K inhibitors induces apoptosis and reduces proliferation [146, 147]. PI3K inhibitors also inhibit the transformation of Rat-1 fibroblasts by NPM-ALK [146], and a dominant negative p85 subunit unable to associate with the p110 subunit was demonstrated to inhibit the ability of NPM-ALK-expressing Ba/F3 cells to form colonies in methylcellulose [147]. Several downstream targets are regulated by PI3K in ALK+ ALCL.

The Akt substrate, glycogen synthase kinase-3*β* (GSK-3*β*), is an important target of NPM-ALK signalling in ALK+ ALCL. Phosphorylation of GSK-3*β* on serine 9 by Akt inhibits GSK-3*β* activity [148], and in ALK+ ALCL this has been argued to be important for preventing GSK-3*β* from phosphorylating, and targeting for degradation, the antiapoptotic protein Mcl-1 and the positive cell cycle-regulator, phosphatase CDC25A [149]. Furthermore, this study showed that phosphorylation of GSK-3*β* on serine 9 correlated with elevated CDC25A levels in ALK+ ALCL patient tumour biopsies. A separate study also demonstrated that NPM-ALK promotes CDC25A expression through PI3K, through either transcriptional upregulation of *CDC25A* or enhanced *CDC25A* mRNA stability [150]. Further supporting the notion that inhibition of GSK-3*β* is an important target of NPM-ALK signalling, treatment of ALK+ ALCL cell lines with either GSK-3*β* shRNA or a GSK-3*β* inhibitor could partially rescue the decreased viability associated with ALK inhibitor treatment [149].

NPM-ALK/PI3K/Akt signalling also activates the sonic hedgehog (SHH) pathway in ALK+ ALCL [151]. SHH is a secreted molecule that, when bound to its receptor Patched, relieves inhibition of the Smoothened co-receptor by Patched. This allows Smoothened to activate glioma-associated homologue (GLI) transcription factors [152]. SHH and GLI1 were found to be highly expressed in primary ALK+ ALCL patient samples, and their expression in cell lines was dependent on NPM-ALK and PI3K activity [151]. It was argued in this study that PI3K-mediated activation of Akt is important for inhibiting GSK-3*β* in order to prevent GSK-3*β* from phosphorylating GLI1 and targeting the protein for proteasomal degradation. NPM-ALK was also found to enhance GLI1 transcriptional activity, and expression of the GLI1 target gene, *cyclin D2 *[151]. Moreover, the inhibition of GLI1 in ALK+ ALCL cell lines, either through siRNA-mediated knockdown or treatment of cells with a Smoothened inhibitor, reduced viability and arrested cells in the G1 stage of the cell cycle [151].

Another target of Akt signalling in ALK+ ALCL is the FOXO3a transcription factor [153]. The phosphorylation of FOXO3a by Akt results in its binding to 14-3-3 proteins, which sequesters FOXO3a in the cytoplasm where it is unable to promote transcription [154]. FOXO3a is phosphorylated in ALK+ ALCL cell lines and in cells ectopically expressing NPM-ALK [153]. Accordingly NPM-ALK signalling results in the down-regulation of the pro-apoptotic protein, Bim-1 and the cell cycle-inhibitor, p27^*kip*1^ [153], which are transcriptional targets of FOXO3a [155, 156]. NPM-ALK/PI3K/Akt signalling also maintains low levels of p27^*kip*1^ by phosphorylating p27^*kip*1^, and thereby targeting p27^*kip*1^ for proteasomal degradation [157, 158].

The activation of the PI3K pathway in ALK+ ALCL is largely dependent on the activity of NPM-ALK. PI3K complexes with NPM-ALK in ALK+ ALCL cell lines [146, 147, 159] and cells isolated from NPM-ALK transgenic mice [92]. Akt is activated in ALK+ ALCL cell lines and patient samples [147]. The activation of Akt in this lymphoma is dependent on NPM-ALK and PI3K activity [126, 127, 160], and Akt activity is upregulated in a PI3K-dependent manner by ectopically expressed NPM-ALK in Ba/F3 cells [127, 146, 153]. PTEN, a lipid phosphatase that dephosphorylates PI3K lipid products [144, 145], is not expressed in some ALK+ ALCL patient samples, and this may be a contributing factor to Akt activation in these patients [161].

## 8. Conclusions and Future Perspectives

It has been over 15 years since the discovery of the NPM-ALK oncoprotein. In this time we have learned much about the signalling pathways activated by NPM-ALK in ALK+ ALCL, and how these pathways contribute to proliferation and survival of this lymphoma. This information has been critical in directing research towards understanding how ALK translocations signal and function in other malignancies. For example, STAT3 activation has been observed in clathrin heavy chain- (CTLC-)ALK-expressing DLBCL patient samples [162], and STAT3, ERK, and AKT are active in EML4-ALK-expressing NSCLC cell lines [163–165]; however, the importance of these pathways in NSCLC and their regulation by EML4-ALK appears to vary amongst NSCLC cell lines [163–165]. Yet, even if activation of the STAT3, ERK, and PI3K/Akt pathways is common to malignancies expressing ALK fusion proteins, differences almost certainly exist in the genes regulated by these pathways in the individual cancers. Some of these differences may be important in the pathogenesis of their respective malignancies. Thus, a more thorough characterization of these signalling pathways in other ALK fusion protein-expressing malignancies needs to be a priority of future research.

While the information gained from elucidating how NPM-ALK signals in ALK+ ALCL has been, and will continue to be, beneficial for understanding how other ALK fusion proteins signal, it is clear that these fusion proteins are not identical in their signalling capability. In a study by Armstrong and colleagues, NIH 3T3 cells expressing the NPM-, Trk-fused gene (TFG)-, 5-aminoimidazole-4-carboxamide ribonucleotide formyltransferase/IMP cyclohydrolase (ATIC)-, tropomyosin 3 (TPM3)-, or CTLC-ALK fusion proteins at roughly equal levels, differed in their ability to activate STAT3 and Akt [166]. The proliferation rate, invasiveness, and ability to form tumours in nude mice also differed amongst the cells expressing the different ALK fusion proteins [166]. Similarly, gene expression profiling demonstrated that, while tumours from ALK+ ALCL patients expressing NPM-ALK or TPM3-ALK shared many commonly regulated genes, distinctly regulated genes were observed [167]. Accordingly, a second focus of future research needs to be a more detailed examination of whether distinctions exist in the signalling pathways or cellular processes regulated by different ALK fusion proteins within the same malignancy.

## Figures and Tables

**Table 1:** Identified ALK fusion proteins and their associated malignancies. Known ALK fusion proteins and the cancers they have been identified in are indicated. ALCL: anaplastic large cell lymphoma; DLBCL: diffuse large B-cell lymphoma; IMT: inflammatory myofibroblastic tumour; NSCLC: nonsmall cell lung carcinoma; RCC: renal cell carcinoma; SCC: squamous cell carcinoma.

Fusion protein	Tumour type	Reference
NPM-ALK	ALCL, DLBCL	[20–22]
TPM3-ALK	ALCL, IMT, RCC	[23–26]
TFG-ALK	ALCL, NSCLC	[27, 28]
ATIC-ALK	ALCL, IMT	[29–32]

CLTC-ALK	ALCL, DLBCL, IMT, extramedullary plasmacytoma	[33–38]

TPM4-ALK	IMT, ALCL, SCC	[25, 39–41]
MSN-ALK	ALCL	[42]
ALO17-ALK	ALCL	[43]
CARS-ALK	IMT	[43]
RANBP2-ALK	IMT	[44]
MYH9-ALK	ALCL	[45]
SEC31L1-ALK	IMT, DLBCL	[46–48]

EML4-ALK	NSCLC, breast, colorectal, RCC	[26, 28, 49, 50]

SQSTM1-ALK	DLBCL	[51]
VCL-ALK	RCC	[52, 53]
KIF5B-ALK	NSCLC	[54]
PPFIBP1-ALK	IMT	[55]
C2orf44-ALK	Colorectal	[56]
KLC1-ALK	NSCLC	[57]

**Table 2:** Known or suspected dimerization/oligomerization domains in ALK fusion partners. Dimerization/oligomerization domains present ALK fusion partners that are postulated to mediate dimerization/oligomerization are indicated. With the exception of the basic domain of EML4-ALK, these domains have not been experimentally proven to mediate dimerization/oligomerization of the respective fusion proteins. The basic domain of EML4 also possesses a coiled-coil motif which is postulated to mediate dimerization.

Dimerization/oligomerization domain	Fusion protein	Reference
Coiled-coil	TPM3-ALK	[23, 25]
	TPM4-ALK	[25]
	TFG-ALK	[27]
	KIF5B-ALK	[54]
	PPFIBP1-ALK	[55]
	MYH9-ALK^∗^	[45]

Leucine zipper	RANBP2-ALK	[44]
Basic domain/coiled-coil	EML4-ALK	[49]
PB1 domain	SQSTM1-ALK	[51]
WD40 repeats	SEC31L1-ALK	[46]
Triskelion assembly motifs	CLTC-ALK	[33]

^∗^ The MYH9 coiled-coil domain is truncated in the fusion protein and may not be functional.

## References

[1] Pulford K, Lamant L, Morris SW (1997). Detection of anaplastic lymphoma kinase (ALK) and nucleolar protein nucleophosmin (NPM)-ALK proteins in normal and neoplastic cells with the monoclonal antibody ALK1. *Blood*.

[2] Iwahara T, Fujimoto J, Wen D (1997). Molecular characterization of ALK, a receptor tyrosine kinase expressed specifically in the nervous system. *Oncogene*.

[3] Morris SW, Naeve C, Mathew P (1997). ALK the chromosome 2 gene locus altered by the t(2;5) in non-Hodgkin’s lymphoma, encodes a novel neural receptor tyrosine kinase that is highly related to leukocyte tyrosine kinase (LTK). *Oncogene*.

[4] Vernersson E, Khoo NKS, Henriksson ML, Roos G, Palmer RH, Hallberg B (2006). Characterization of the expression of the ALK receptor tyrosine kinase in mice. *Gene Expression Patterns*.

[5] Palmer RH, Vernersson E, Grabbe C, Hallberg B (2009). Anaplastic lymphoma kinase: signalling in development and disease. *Biochemical Journal*.

[6] Weiss JB, Xue C, Benice T, Xue L, Morris SW, Raber J (2012). Anaplastic lymphoma kinase and leukocyte tyrosine kinase: functions and genetic interactions in learning, memory and adult neurogenesis. *Pharmacology, Biochemistry, and Behavior*.

[7] Bilsland JG, Wheeldon A, Mead A (2008). Behavioral and neurochemical alterations in mice deficient in anaplastic lymphoma kinase suggest therapeutic potential for psychiatric indications. *Neuropsychopharmacology*.

[8] Lasek AW, Lim J, Kliethermes CL (2011). An evolutionary conserved role for anaplastic lymphoma kinase in behavioral responses to ethanol. *PLoS ONE*.

[9] Kunugi H, Hashimoto R, Okada T (2006). Possible association between nonsynonymous polymorphisms of the anaplastic lymphoma kinase (ALK) gene and schizophrenia in a Japanese population. *Journal of Neural Transmission*.

[10] Englund C, Lorén CE, Grabbe C (2003). Jeb signals through the Alk receptor tyrosine kinase to drive visceral muscle fusion. *Nature*.

[11] Lee HH, Norris A, Weiss JB, Frasch M (2003). Jelly belly protein activates the receptor tyrosine kinase Alk to specify visceral muscle pioneers. *Nature*.

[12] Stute C, Schimmelpfeng K, Renkawitz-Pohl R, Palmer RH, Holz A (2004). Myoblast determination in the somatic and visceral mesoderm depends on Notch signalling as well as on milliways (miliAlk) as receptor for jeb signalling. *Development*.

[13] Stoica GE, Kuo A, Aigner A (2001). Identification of anaplastic lymphoma kinase as a receptor for the growth factor pleiotrophin. *Journal of Biological Chemistry*.

[14] Stoica GE, Kuo A, Powers C (2002). Midkine binds to anaplastic lymphoma kinase (ALK) and acts as a growth factor for different cell types. *Journal of Biological Chemistry*.

[15] Moog-Lutz C, Degoutin J, Gouzi JY (2005). Activation and inhibition of anaplastic lymphoma kinase receptor tyrosine kinase by monoclonal antibodies and absence of agonist activity of pleiotrophin. *Journal of Biological Chemistry*.

[16] Motegi A, Fujimoto J, Kotani M, Sakuraba H, Yamamoto T (2004). ALK receptor tyrosine kinase promotes cell growth and neurite outgrowth. *Journal of Cell Science*.

[17] Perez-Pinera P, Zhang W, Chang Y, Vega JA, Deuel TF (2007). Anaplastic lymphoma kinase is activated through the pleiotrophin/receptor protein-tyrosine phosphatase *β*/*ζ* signaling pathway: an alternative mechanism of receptor tyrosine kinase activation. *Journal of Biological Chemistry*.

[18] Mourali J, Bénard A, Lourenço FC (2006). Anaplastic lymphoma kinase is a dependence receptor whose proapoptotic functions are activated by caspase cleavage. *Molecular and Cellular Biology*.

[19] Mehlen P, Bredesen DE (2004). The dependence receptor hypothesis. *Apoptosis*.

[20] Morris SW, Kirstein MN, Valentine MB (1994). Fusion of a kinase gene, ALK, to a nucleolar protein gene, NPM, in non-Hodgkin’s lymphoma. *Science*.

[21] Shiota M, Fujimoto J, Semba T, Satoh H, Yamamoto T, Mori S (1994). Hyperphosphorylation of a novel 80 kDa protein-tyrosine kinase similar to Ltk in a human Ki-1 lymphoma cell line, AMS3. *Oncogene*.

[22] Arber DA, Sun LH, Weiss LM (1996). Detection of the t(2;5)(p23;q35) chromosomal translocation in large B- cell lymphomas other than anaplastic large cell lymphoma. *Human Pathology*.

[23] Lamant L, Dastugue N, Pulford K, Delsol G, Mariamé B (1999). A new fusion gene TPM3-ALK in anaplastic large cell lymphoma created by a (1;2)(q25;p23) translocation. *Blood*.

[24] Siebert R, Gesk S, Harder L (1999). Complex variant translocation t(1;2) with TPM3-ALK fusion due to cryptic ALK gene rearrangement in anaplastic large-cell lymphoma. *Blood*.

[25] Lawrence B, Perez-Atayde A, Hibbard MK (2000). TPM3-ALK and TPM4-ALK oncogenes in inflammatory myofibroblastic tumors. *American Journal of Pathology*.

[26] Sugawara E, Togashi Y, Kuroda N Identification of anaplastic lymphoma kinase fusions in renal cancer: large-scale immunohistochemical screening by the intercalated antibody-enhanced polymer method.

[27] Hernández L, Pinyol M, Hernández S (1999). TRK-fused gene (TFG) is a new partner of ALK in anaplastic large cell lymphoma producing two structurally different TFG-ALK translocations. *Blood*.

[28] Rikova K, Guo A, Zeng Q (2007). Global survey of phosphotyrosine signaling identifies oncogenic kinases in lung cancer. *Cell*.

[29] Trinei M, Lanfrancone L, Campo E (2000). A new variant anaplastic lymphoma kinase (ALK)-fusion protein (ATIC-ALK) in a case of ALK-positive anaplastic large cell lymphoma. *Cancer Research*.

[30] Ma Z, Cools J, Marynen P (2000). Inv(2)(p23q35) in anaplastic large-cell lymphoma induces constitutive anaplastic lymphoma kinase (ALK) tyrosine kinase activation by fusion to ATIC, an enzyme involved in purine nucleotide biosynthesis. *Blood*.

[31] Colleoni GWB, Bridge JA, Garicochea B, Liu J, Filippa DA, Ladanyi M (2000). ATIC-ALK: a novel variant ALK gene fusion in anaplastic large cell lymphoma resulting from the recurrent cryptic chromosomal inversion, inv(2)(p23q35). *American Journal of Pathology*.

[32] Debiec-Rychter M, Marynen P, Hagemeijer A, Pauwels P (2003). ALK-ATIC fusion in urinary bladder inflammatory myofibroblastic tumor. *Genes Chromosomes and Cancer*.

[33] Touriol C, Greenland C, Lamant L (2000). Further demonstration of the diversity of chromosomal changes involving 2p23 in ALK-positive lymphoma: 2 cases expressing ALK kinase fused to CLTCL (clathrin chain polypeptide-like). *Blood*.

[34] Bridge JA, Kanamori M, Ma Z (2001). Fusion of the ALK gene to the clathrin heavy chain gene, CLTC, in inflammatory myofibroblastic tumor. *American Journal of Pathology*.

[35] De Paepe P, Baens M, van Krieken H (2003). ALK activation by the CLTC-ALK fusion is a recurrent event in large B-cell lymphoma. *Blood*.

[36] Gascoyne RD, Lamant L, Martin-Subero JI (2003). ALK-positive diffuse large B-cell lymphoma is associated with Clathrin-ALK rearrangements: report of 6 cases. *Blood*.

[37] Chikatsu N, Kojima H, Suzukawa K (2003). ALK^+^, CD30^−^, CD20^−^ large B-cell lymphoma containing anaplastic lymphoma kinase (ALK) fused to clathrin heavy chain gene (CLTC). *Modern Pathology*.

[38] Wang WY, Gu L, Liu WP, Li GD, Liu HJ, Ma ZG (2011). ALK-positive extramedullary plasmacytoma with expression of the CLTC-ALK fusion transcript. *Pathology, Research and Practice*.

[39] Meech SJ, McGavran L, Odom LF (2001). Unusual childhood extramedullary hematologic malignancy with natural killer cell properties that contains tropomyosin 4—anaplastic lymphoma kinase gene fusion. *Blood*.

[40] Jazii FR, Najafi Z, Malekzadeh R (2006). Identification of squamous cell carcinoma associated proteins by proteomics and loss of beta tropomyosin expression in esophageal cancer. *World Journal of Gastroenterology*.

[41] Du XL, Hu H, Lin DC (2007). Proteomic profiling of proteins dysregulted in Chinese esophageal squamous cell carcinoma. *Journal of Molecular Medicine*.

[42] Tort F, Pinyol M, Pulford K (2001). Molecular characterization of a new ALK translocation involving moesin (MSN-ALK) in anaplastic large cell lymphoma. *Laboratory Investigation*.

[43] Cools J, Wlodarska I, Somers R (2002). Identification of novel fusion partners of ALK, the anaplastic lymphoma kinase, in anaplastic large-cell lymphoma and inflammatory myofibroblastic tumor. *Genes Chromosomes and Cancer*.

[44] Ma Z, Hill DA, Collins MH (2003). Fusion of ALK to the Ran-binding protein 2 (RANBP2) gene in inflammatory myofibroblastic tumor. *Genes Chromosomes and Cancer*.

[45] Lamant L, Gascoyne RD, Duplantier MM (2003). Non-muscle myosin heavy chain (MYH9): a new partner fused to ALK in anaplastic large cell lymphoma. *Genes Chromosomes and Cancer*.

[46] Panagopoulos I, Nilsson T, Domanski HA (2006). Fusion of the SEC31L1 and ALK genes in an inflammatory myofibroblastic tumor. *International Journal of Cancer*.

[47] Van Roosbroeck K, Cools J, Dierickx D (2010). ALK-positive large B-cell lymphomas with cryptic SEC31A-ALK and NPM1-ALK fusions. *Haematologica*.

[48] Bedwell C, Rowe D, Moulton D, Jones G, Bown N, Bacon CM (2011). Cytogenetically complex SEC31A-ALK fusions are recurrent in ALK-positive large B-cell lymphomas. *Haematologica*.

[49] Soda M, Choi YL, Enomoto M (2007). Identification of the transforming EML4-ALK fusion gene in non-small-cell lung cancer. *Nature*.

[50] Lin E, Li L, Guan Y (2009). Exon array profiling detects EML4-ALK fusion in breast, colorectal, and non-small cell lung cancers. *Molecular Cancer Research*.

[51] Takeuchi K, Soda M, Togashi Y (2011). Identification of a novel fusion, SQSTM1-ALK, in ALK-positive large B-cell lymphoma. *Haematologica*.

[52] Debelenko LV, Raimondi SC, Daw N (2011). Renal cell carcinoma with novel VCL-ALK fusion: new representative of ALK-associated tumor spectrum. *Modern Pathology*.

[53] Mariño-Enríquez A, Ou WB, Weldon CB, Fletcher JA, Pérez-Atayde AR (2011). ALK rearrangement in sickle cell trait-associated renal medullary carcinoma. *Genes Chromosomes and Cancer*.

[54] Takeuchi K, Young LC, Togashi Y (2009). KIF5B-ALK, a novel fusion oncokinase identified by an immunohistochemistry-based diagnostic system for ALK-positive lung cancer. *Clinical Cancer Research*.

[55] Takeuchi K, Soda M, Togashi Y (2011). Pulmonary inflammatory myofibroblastic tumor expressing a novel fusion, PPFIBP1-ALK: reappraisal of anti-ALK immunohistochemistry as a tool for novel ALK fusion identification. *Clinical Cancer Research*.

[56] Lipson D, Capelletti M, Yelensky R (2012). Identification of new ALK and RET gene fusions from colorectal and lung cancer biopsies. *Nature Medicine*.

[57] Togashi Y, Soda M, Sakata S (2012). KLC1-ALK: a novel fusion in lung cancer identified using a formalin-fixed paraffin-embedded tissue only. *PLoS ONE*.

[58] Delsol G, Falini B, Muller-Hermelink HK (2008). *Anaplastic Large Cell Lymphoma (ALCL), ALK-Positive*.

[59] Fornari A, Piva R, Chiarle R, Novero D, Inghirami G (2009). Anaplastic large cell lymphoma: one or more entities among T-cell lymphoma?. *Hematological Oncology*.

[60] Fischer P, Nacheva E, Mason DY (1988). A Ki-1 (CD30)-positive human cell line (Karpas 299) established from a high grade non-Hodgkin’s lymphoma, showing a 2;5 translocation and rearrangement of the T-cell receptor *β*-chain gene. *Blood*.

[61] Rimokh R, Magaud JP, Berger F (1989). A translocation involving a specific breakpoint (q35) on chromosome 5 is characteristic of anaplastic large cell lymphoma (Ki-1 lymphoma). *British Journal of Haematology*.

[62] Kaneko Y, Frizzera G, Edamura S (1989). A novel translocation, t(2;5)(p23;q35), in childhood phagocytic large T-cell lymphoma mimicking malignant histiocytosis. *Blood*.

[63] Mason DY, Bastard C, Rimokh R (1990). CD30-positive large cell lymphomas (Ki-1 lymphoma) are associated with a chromosomal translocation involving 5q35. *British Journal of Haematology*.

[64] Le Beau MM, Bitter MA, Larson RA (1989). The t(2;5)(p23;q35): a recurring chromosomal abnormality in Ki-1-positive anaplastic large cell lymphoma. *Leukemia*.

[65] Griffin CA, Hawkins AL, Dvorak C, Henkle C, Ellingham T, Perlman EJ (1999). Recurrent involvement of 2p23 in inflammatory myofibroblastic tumors. *Cancer Research*.

[66] Gambacorti-Passerini C, Messa C, Pogliani EM (2011). Crizotinib in anaplastic large-cell lymphoma. *The New England Journal of Medicine*.

[67] Kwak EL, Bang YJ, Camidge DR (2010). Anaplastic lymphoma kinase inhibition in non-small-cell lung cancer. *The New England Journal of Medicine*.

[68] Butrynski JE, D’Adamo DR, Hornick JL (2010). Crizotinib in ALK-rearranged inflammatory myofibroblastic tumor. *The New England Journal of Medicine*.

[69] Perez-Pinera P, Chang Y, Astudillo A, Mortimer J, Deuel TF (2007). Anaplastic lymphoma kinase is expressed in different subtypes of human breast cancer. *Biochemical and Biophysical Research Communications*.

[70] Lamant L, Pulford K, Bischof D (2000). Expression of the ALK tyrosine kinase gene in neuroblastoma. *American Journal of Pathology*.

[71] Janoueix-Lerosey I, Lequin D, Brugières L (2008). Somatic and germline activating mutations of the ALK kinase receptor in neuroblastoma. *Nature*.

[72] Chen Y, Takita J, Choi YL (2008). Oncogenic mutations of ALK kinase in neuroblastoma. *Nature*.

[73] George RE, Sanda T, Hanna M (2008). Activating mutations in ALK provide a therapeutic target in neuroblastoma. *Nature*.

[74] Carén H, Abel F, Kogner P, Martinsson T (2008). High incidence of DNA mutations and gene amplifications of the ALK gene in advanced sporadic neuroblastoma tumours. *Biochemical Journal*.

[75] Mossé YP, Laudenslager M, Longo L (2008). Identification of ALK as a major familial neuroblastoma predisposition gene. *Nature*.

[76] Murugan AK, Xing MM (2011). Anaplastic thyroid cancers harbor novel oncogenic mutations of the ALK gene. *Cancer Research*.

[77] Wang D, Umekawa H, Olson MOJ (1993). Expression and subcellular locations of two forms of nucleolar protein B23 in rat tissues and cells. *Cellular and Molecular Biology Research*.

[78] Borer RA, Lehner CF, Eppenberger HM, Nigg EA (1989). Major nucleolar proteins shuttle between nucleus and cytoplasm. *Cell*.

[79] Colombo E, Alcalay M, Pelicci PG (2011). Nucleophosmin and its complex network: a possible therapeutic target in hematological diseases. *Oncogene*.

[80] Fujimoto J, Shiota M, Iwahara T (1996). Characterization of the transforming activity of p80, a hyperphosphorylated protein in a Ki-1 lymphoma cell line with chromosomal translocation t(2;5). *Proceedings of the National Academy of Sciences of the United States of America*.

[81] Bischof D, Pulford K, Mason DY, Morris SW (1997). Role of the nucleophosmin (NPM) portion of the non-Hodgkin’s lymphoma- associated NPM-anaplastic lymphoma kinase fusion protein in oncogenesis. *Molecular and Cellular Biology*.

[82] Chan PK (1989). Cross-linkage of nucleophosmin in tumor cells by nitrogen mustard. *Cancer Research*.

[83] Yung BYM, Chan PK (1987). Identification and characterization of a hexameric form of nucleolar phosphoprotein B23. *Biochimica et Biophysica Acta*.

[84] Mano H (2008). Non-solid oncogenes in solid tumors: EML4-ALK fusion genes in lung cancer. *Cancer Science*.

[85] Barreca A, Lasorsa E, Riera L (2011). Anaplastic lymphoma kinase in human cancer. *Journal of Molecular Endocrinology*.

[86] Chiarle R, Voena C, Ambrogio C, Piva R, Inghirami G (2008). The anaplastic lymphoma kinase in the pathogenesis of cancer. *Nature Reviews Cancer*.

[87] Levy DE, Darnell JE (2002). STATs: transcriptional control and biological impact. *Nature Reviews Molecular Cell Biology*.

[88] Santos CI, Costa-Pereira AP (2011). Signal transducers and activators of transcription-from cytokine signalling to cancer biology. *Biochimica et Biophysica Acta*.

[89] Zhang Q, Raghunath PN, Xue L (2002). Multilevel dysregulation of STAT3 activation in anaplastic lymphoma kinase-positive T/null-cell lymphoma. *Journal of Immunology*.

[90] Zamo A, Chiarle R, Piva R (2002). Anaplastic lymphoma kinase (ALK) activates Stat3 and protects hematopoietic cells from cell death. *Oncogene*.

[91] Khoury JD, Medeiros LJ, Rassidakis GZ (2003). Differential expression and clinical significance of tyrosine-phosphorylated STAT3 in ALK^+^ and ALK^−^ anaplastic large cell lymphoma. *Clinical Cancer Research*.

[92] Chiarle R, Gong JZ, Guasparri I (2003). NPM-ALK transgenic mice spontaneously develop T-cell lymphomas and plasma cell tumors. *Blood*.

[93] Chiarle R, Simmons WJ, Cai H (2005). Stat3 is required for ALK-mediated lymphomagenesis and provides a possible therapeutic target. *Nature Medicine*.

[94] Amin HM, McDonnell TJ, Ma Y (2004). Selective inhibition of STAT3 induces apoptosis and G1 cell cycle arrest in ALK-positive anaplastic large cell lymphoma. *Oncogene*.

[95] Piva R, Agnelli L, Pellegrino E (2010). Gene expression profiling uncovers molecular classifiers for the recognition of anaplastic large-cell lymphoma within peripheral T-cell neoplasms. *Journal of Clinical Oncology*.

[96] Zhang Q, Wang H, Kantekure K (2011). Oncogenic tyrosine kinase NPM-ALK induces expression of the growth-promoting receptor ICOS. *Blood*.

[97] Anastasov N, Bonzheim I, Rudelius M (2010). C/EBP*β* expression in ALK-positiveanaplastic large cell lymphomas is required for cell proliferation and is induced by the STAT3 signaling pathway. *Haematologica*.

[98] Piva R, Pellegrino E, Mattioli M (2006). Functional validation of the anaplastic lymphoma kinase signature identifies CEBPB and BCl2A1 as critical target genes. *Journal of Clinical Investigation*.

[99] Marzec M, Zhang Q, Goradia A (2008). Oncogenic kinase NPM/ALK induces through STAT3 expression of immunosuppressive protein CD274 (PD-L1, B7-H1). *Proceedings of the National Academy of Sciences of the United States of America*.

[100] Yamamoto R, Nishikori M, Tashima M (2009). B7-H1 expression is regulated by MEK/ERK signaling pathway in anaplastic large cell lymphoma and Hodgkin lymphoma. *Cancer Science*.

[101] Lai R, Rassidakis GZ, Medeiros LJ (2004). Signal transducer and activator of transcription-3 activation contributes to high tissue inhibitor of metalloproteinase-1 expression in anaplastic lymphoma kinase-positive anaplastic large cell lymphoma. *American Journal of Pathology*.

[102] Marzec M, Liu X, Wong W (2011). Oncogenic kinase NPM/ALK induces expression of HIF1*α* mRNA. *Oncogene*.

[103] Zhang J, Wang P, Wu F (2012). Aberrant expression of the transcriptional factor Twist1 promotes invasiveness in ALK-positive anaplastic large cell lymphoma. *Cellular Signalling*.

[104] Kasprzycka M, Marzec M, Liu X, Zhang Q, Wasik MA (2006). Nucleophosmin/anaplastic lymphoma kinase (NPM/ALK) oncoprotein induces the T regulatory cell phenotype by activating STAT3. *Proceedings of the National Academy of Sciences of the United States of America*.

[105] Ambrogio C, Martinengo C, Voena C (2009). NPM-ALK oncogenic tyrosine kinase controls T-cell identity by transcriptional regulation and epigenetic silencing in lymphoma cells. *Cancer Research*.

[106] Sasai N, Defossez PA (2009). Many paths to one goal? The proteins that recognize methylated DNA in eukaryotes. *The International Journal of Developmental Biology*.

[107] Zhang Q, Wang HY, Liu X, Bhutani G, Kantekure K, Wasik M (2011). IL-2R common *γ*-chain is epigenetically silenced by nucleophosphin-anaplastic lymphoma kinase (NPM-ALK) and acts as a tumor suppressor by targeting NPM-ALK. *Proceedings of the National Academy of Sciences of the United States of America*.

[108] Zhang Q, Wang HY, Liu X, Wasik MA (2007). STAT5A is epigenetically silenced by the tyrosine kinase NPM1-ALK and acts as a tumor suppressor by reciprocally inhibiting NPM1-ALK expression. *Nature Medicine*.

[109] Crockett DK, Lin Z, Elenitoba-Johnson KSJ, Lim MS (2004). Identification of NPM-ALK interacting proteins by tandem mass spectrometry. *Oncogene*.

[110] Amin HM, Medeiros LJ, Ma Y (2003). Inhibition of JAK3 induces apoptosis and decreases anaplastic lymphoma kinase activity in anaplastic large cell lymphoma. *Oncogene*.

[111] Marzec M, Kasprzycka M, Ptasznik A (2005). Inhibition of ALK enzymatic activity in T-cell lymphoma cells induces apoptosis and suppresses proliferation and STAT3 phosphorylation independently of Jak3. *Laboratory Investigation*.

[112] Khoury JD, Rassidakis GZ, Medeiros LJ, Amin HM, Lai R (2004). Methylation of SHP1 gene and loss of SHP1 protein expression are frequent in systemic anaplastic large cell lymphoma. *Blood*.

[113] Zhang Q, Wang HY, Marzec M, Raghunath PN, Nagasawa T, Wasik MA (2005). STAT3- and DNA methyltransferase 1-mediated epigenetic silencing of SHP-1 tyrosine phosphatase tumor suppressor gene in malignant T lymphocytes. *Proceedings of the National Academy of Sciences of the United States of America*.

[114] Honorat JF, Ragab A, Lamant L, Delsol G, Ragab-Thomas J (2006). SHP1 tyrosine phosphatase negatively regulates NPM-ALK tyrosine kinase signaling. *Blood*.

[115] Han Y, Amin HM, Franko B, Frantz C, Shi X, Lai R (2006). Loss of SHP1 enhances JAK3/STAT3 signaling and decreases proteosome degradation of JAK3 and NPM-ALK in ALK^+^ anaplastic large-cell lymphoma. *Blood*.

[116] Han Y, Amin HM, Frantz C (2006). Restoration of shp1 expression by 5-AZA-2′-deoxycytidine is associated with downregulation of JAK3/STAT3 signaling in ALK-positive anaplastic large cell lymphoma. *Leukemia*.

[117] Qiu L, Lai R, Lin Q (2006). Autocrine release of interleukin-9 promotes Jak3-dependent survival of ALK^+^ anaplastic large-cell lymphoma cells. *Blood*.

[118] Bard JD, Gelebart P, Anand M (2009). IL-21 contributes to JAK3/STAT3 activation and promotes cell growth in ALK-positive anaplastic large cell lymphoma. *American Journal of Pathology*.

[119] Bard JD, Gelebart P, Anand M, Amin HM, Lai R (2008). Aberrant expression of IL-22 receptor 1 and autocrine IL-22 stimulation contribute to tumorigenicity in ALK^+^ anaplastic large cell lymphoma. *Leukemia*.

[120] Geest CR, Coffer PJ (2009). MAPK signaling pathways in the regulation of hematopoiesis. *Journal of Leukocyte Biology*.

[121] Steelman LS, Franklin RA, Abrams SL (2011). Roles of the Ras/Raf/MEK/ERK pathway in leukemia therapy. *Leukemia*.

[122] Watanabe M, Sasaki M, Itoh K (2005). JunB induced by constitutive CD30-extracellular signal-regulated kinase 1/2 mitogen-activated protein kinase signaling activates the CD30 promoter in anaplastic large cell lymphoma and Reed-Sternberg cells of Hodgkin lymphoma. *Cancer Research*.

[123] Staber PB, Vesely P, Haq N (2007). The oncoprotein NPM-ALK of anaplastic large-cell lymphoma induces JUNB transcription via ERK1/2 and JunB translation via mTOR signaling. *Blood*.

[124] Marzec M, Kasprzycka M, Liu X, Raghunath PN, Wlodarski P, Wasik MA (2007). Oncogenic tyrosine kinase NPM/ALK induces activation of the MEK/ERK signaling pathway independently of c-Raf. *Oncogene*.

[125] Lim MS, Carlson ML, Crockett DK (2009). The proteomic signature of NPM/ALK reveals deregulation of multiple cellular pathways. *Blood*.

[126] Vega F, Medeiros LJ, Leventaki V (2006). Activation of mammalian target of rapamycin signaling pathway contributes to tumor cell survival in anaplastic lymphoma kinase-positive anaplastic large cell lymphoma. *Cancer Research*.

[127] Marzec M, Kasprzycka M, Liu X (2007). Oncogenic tyrosine kinase NPM/ALK induces activation of the rapamycin-sensitive mTOR signaling pathway. *Oncogene*.

[128] Magnuson B, Ekim B, Fingar DC (2012). Regulation and function of ribosomal protein S6 kinase (S6K) within mTOR signalling networks. *Biochemical Journal*.

[129] Fernandez M, Manso R, Bernaldez F, Lopez P, Martin-Duce A, Alemany S (2011). Involvement of Cot activity in the proliferation of ALCL lymphoma cells. *Biochemical and Biophysical Research Communications*.

[130] Manning BD, Cantley LC (2003). Rheb fills a GAP between TSC and TOR. *Trends in Biochemical Sciences*.

[131] Ma XM, Blenis J (2009). Molecular mechanisms of mTOR-mediated translational control. *Nature Reviews Molecular Cell Biology*.

[132] Merkel O, Hamacher F, Laimer D (2010). Identification of differential and functionally active miRNAs in both anaplastic lymphoma kinase (ALK)^+^ and ALK^−^ anaplastic large-cell lymphoma. *Proceedings of the National Academy of Sciences of the United States of America*.

[133] Watanabe M, Itoh K, Togano T, Kadin ME, Watanabe T, Horie R (2012). Ets-1 activates overexpression of JunB and CD30 in Hodgkin's lymphoma and anaplastic large-cell lymphoma. *The American Journal of Pathology*.

[134] Mathas S, Hinz M, Anagnostopoulos I (2002). Aberrantly expressed c-Jun and JunB are a hallmark of Hodgkin lymphoma cells, stimulate proliferation and synergize with NF-*κ*B. *The EMBO Journal*.

[135] Szremska AP, Kenner L, Weisz E (2003). JunB inhibits proliferation and transformation in B-lymphoid cells. *Blood*.

[136] Rassidakis GZ, Thomaides A, Atwell C (2005). JunB expression is a common feature of CD30^+^ lymphomas and lymphomatoid papulosis. *Modern Pathology*.

[137] Hsu FYY, Johnston PB, Burke KA, Zhao Y (2006). The expression of CD30 in anaplastic large cell lymphoma is regulated by nucleophosmin-anaplastic lymphoma kinase-mediated JunB level in a cell type-specific manner. *Cancer Research*.

[138] Pearson JD, Lee JK, Bacani JT, Lai R, Ingham RJ (2011). NPM-ALK and the JunB transcription factor regulate the expression of cytotoxic molecules in ALK-positive, anaplastic large cell lymphoma. *International Journal of Clinical and Experimental Pathology*.

[139] Christensen JG, Zou HY, Arango ME (2007). Cytoreductive antitumor activity of PF-2341066, a novel inhibitor of anaplastic lymphoma kinase and c-Met, in experimental models of anaplastic large-cell lymphoma. *Molecular Cancer Therapeutics*.

[140] Ritter U, Damm-Welk C, Fuchs U, Bohle RM, Borkhardt A, Woessmann W (2003). Design and evaluation of chemically synthesized siRNA targeting the NPM-ALK fusion site in anaplastic large cell lymphoma (ALCL). *Oligonucleotides*.

[141] Turner SD, Yeung D, Hadfield K, Cook SJ, Alexander DR (2007). The NPM-ALK tyrosine kinase mimics TCR signalling pathways, inducing NFAT and AP-1 by RAS-dependent mechanisms. *Cellular Signalling*.

[142] Voena C, Conte C, Ambrogio C (2007). The tyrosine phosphatase Shp2 interacts with NPM-ALK and regulates anaplastic lymphoma cell growth and migration. *Cancer Research*.

[143] Riera L, Lasorsa E, Ambrogio C, Surrenti N, Voena C, Chiarle R (2010). Involvement of Grb2 adaptor protein in nucleophosmin-anaplastic lymphoma kinase (NPM-ALK)-mediated signaling and anaplastic large cell lymphoma growth. *Journal of Biological Chemistry*.

[144] Cantley LC (2002). The phosphoinositide 3-kinase pathway. *Science*.

[145] Liu P, Cheng H, Roberts TM, Zhao JJ (2009). Targeting the phosphoinositide 3-kinase pathway in cancer. *Nature Reviews Drug Discovery*.

[146] Bai RY, Ouyang T, Miething C, Morris SW, Peschel C, Duyster J (2000). Nucleophosmin-anaplastic lymphoma kinase associated with anaplastic large-cell lymphoma activates the phosphatidylinositol 3-kinase/Akt antiapoptotic signaling pathway. *Blood*.

[147] Slupianek A, Nieborowska-Skorska M, Hoser G (2001). Role of phosphatidylinositol 3-kinase-Akt pathway in nucleophosmin/anaplastic lymphoma kinase-mediated lymphomagenesis. *Cancer Research*.

[148] Cross DAE, Alessi DR, Cohen P, Andjelkovich M, Hemmings BA (1995). Inhibition of glycogen synthase kinase-3 by insulin mediated by protein kinase B. *Nature*.

[149] McDonnell SR, Hwang SR, Basrur V NPM-ALK signals through glycogen synthase kinase 3beta to promote oncogenesis.

[150] Fernandez-Vidal A, Mazars A, Gautier EF, Prévost G, Payrastre B, Manenti S (2009). Upregulation of the CDC25A phosphatase down-stream of the NPM/ALK oncogene participates to anaplastic large cell lymphoma enhanced proliferation. *Cell Cycle*.

[151] Singh RR, Cho-Vega JH, Davuluri Y (2009). Sonic hedgehog signaling pathway is activated in ALK-positive anaplastic large cell lymphoma. *Cancer Research*.

[152] Zhu H, Lo HW (2010). The human glioma-associated oncogene homolog 1 (GLI1) family of transcription factors in gene regulation and diseases. *Current Genomics*.

[153] Gu TL, Tothova Z, Scheijen B, Griffin JD, Gilliland DG, Sternberg DW (2004). NPM-ALK fusion kinase of anaplastic large-cell lymphoma regulates survival and proliferative signaling through modulation of FOXO3a. *Blood*.

[154] Huang H, Tindall DJ (2007). Dynamic FoxO transcription factors. *Journal of Cell Science*.

[155] Dijkers PF, Medema RH, Lammers JWJ, Koenderman L, Coffer PJ (2000). Expression of the pro-apoptotic Bcl-2 family member Bim is regulated by the forkhead transcription factor FKHR-L1. *Current Biology*.

[156] Dijkers PF, Medema RH, Pals C (2000). Forkhead transcription factor FKHR-L1 modulates cytokine-dependent transcriptional regulation of p27^KIP1^. *Molecular and Cellular Biology*.

[157] Slupianek A, Skorski T (2004). NPM/ALK downregulates p27^KIP1^ in a PI-3K-dependent manner. *Experimental Hematology*.

[158] Rassidakis GZ, Feretzaki M, Atwell C (2005). Inhibition of Akt increases p27^KIP1^ levels and induces cell cycle arrest in anaplastic large cell lymphoma. *Blood*.

[159] Polgar D, Leisser C, Maier S (2005). Truncated ALK derived from chromosomal translocation t(2;5)(p23;q35) binds to the SH3 domain of p85-PI3K. *Mutation Research*.

[160] Wan W, Albom MS, Lu L (2006). Anaplastic lymphoma kinase activity is essential for the proliferation and survival of anaplastic large-cell lymphoma cells. *Blood*.

[161] Uner AH, Saglam A, Han U, Hayran M, Sungur A, Ruacan S (2005). PTEN and p27 expression in mature T-cell and NK-cell neoplasms. *Leukemia and Lymphoma*.

[162] Momose S, Tamaru JI, Kishi H (2009). Hyperactivated STAT3 in ALK-positive diffuse large B-cell lymphoma with clathrin-ALK fusion. *Human Pathology*.

[163] Chen Z, Sasaki T, Tan X (2010). Inhibition of ALK, PI3K/MEK, and HSP90 in murine lung adenocarcinoma induced by EML4-ALK fusion oncogene. *Cancer Research*.

[164] Li Y, Ye X, Liu J, Zha J, Pei L (2011). Evaluation of eml4-alk fusion proteins in non-small cell lung cancer using small molecule inhibitors. *Neoplasia*.

[165] Takezawa K, Okamoto I, Nishio K, Jänne PA, Nakagawa K (2011). Role of ERK-BIM and STAT3-survivin signaling pathways in ALK inhibitor-induced apoptosis in EML4-ALK-positive lung cancer. *Clinical Cancer Research*.

[166] Armstrong F, Duplantier MM, Trempat P (2004). Differential effects of X-ALK fusion proteins on proliferation, transformation, and invasion properties of NIH3T3 cells. *Oncogene*.

[167] Bohling SD, Jenson SD, Crockett DK, Schumacher JA, Elenitoba-Johnson KSJ, Lim MS (2008). Analysis of gene expression profile of TPM3-ALK positive anaplastic large cell lymphoma reveals overlapping and unique patterns with that of NPM-ALK positive anaplastic large cell lymphoma. *Leukemia Research*.

